# Cardiac Effects of Modern Breast Radiation Therapy in Patients Receiving Systemic Cancer Therapy

**DOI:** 10.1016/j.jaccao.2025.01.012

**Published:** 2025-03-04

**Authors:** Eva Berlin, Kyunga Ko, Lin Ma, Ian Messing, Casey Hollawell, Amanda M. Smith, Neil K. Taunk, Vivek Narayan, Jenica N. Upshaw, Amy S. Clark, Payal D. Shah, Hayley Knollman, Saveri Bhattacharya, Daniel Koropeckyj-Cox, Jessica Wang, Nikhil Yegya-Raman, Ivy S. Han, Benedicte Lefebvre, Tang Li, Nicholas S. Wilcox, Wonyoung Jung, Jinbo Chen, Gary M. Freedman, Bonnie Ky

**Affiliations:** a Department of Radiation Oncology, Perelman School of Medicine, University of Pennsylvania, Philadelphia, Pennsylvania, USA; b Division of Cardiology, Department of Medicine, Perelman School of Medicine, University of Pennsylvania, Philadelphia, Pennsylvania, USA; c Department of Radiation Oncology, Mayo Clinic Comprehensive Cancer Center, Rochester, Minnesota, USA; d Division of Hematology and Oncology, Department of Medicine, Perelman School of Medicine, University of Pennsylvania, Philadelphia, Pennsylvania, USA; e Division of Cardiology, Department of Medicine, Tufts Medical Center, Boston, Massachusetts, USA; f Department of Biostatistics, Epidemiology and Informatics, Perelman School of Medicine, University of Pennsylvania, Philadelphia, Pennsylvania, USA

**Keywords:** breast cancer, cardiac toxicity, cardio-oncology, cardiotoxicity, diastolic function, echocardiography, heart failure with preserved ejection fraction, heart failure with reduced ejection fraction, radiation cardiotoxicity, radiation therapy, radiation physics, ventricular-arterial coupling

## Abstract

**Background:**

Radiation therapy (RT) improves breast cancer outcomes, but cardiac morbidity remains a concern.

**Objectives:**

This study sought to evaluate changes in cardiac function after RT and the relationship between cardiac dose metrics and echocardiography-derived measures of function.

**Methods:**

In a longitudinal cohort study of women with breast cancer, radiation cardiac dose metrics and core lab quantitated echocardiographic measures of cardiac function were evaluated. Dose metrics included the whole heart, left ventricle, right ventricle, and left anterior descending artery (LAD). Echocardiographic measures included left ventricular ejection fraction (LVEF), longitudinal strain, circumferential strain, E/e’ (ratio of early diastolic mitral inflow velocity to early diastolic mitral annular tissue velocity), Ea/Es (ventricular arterial coupling; ratio of effective arterial elastance to end systolic elastance), and right ventricular fractional area change. The mean change in echocardiographic measures over time and the association between cardiac dose metrics and echocardiographic measures were estimated by repeated-measures multivariable linear regression via generalized estimating equations.

**Results:**

The cohort included 303 participants (median age 52 years, 33.3% African American) who received adjuvant RT (2010-2019) with a median mean heart dose of 1.19 Gy (Q1-Q3: 0.75-2.61 Gy), were followed over a median of 5.1 years (Q1-Q3: 3.2-7.1 years). Across all participants, there was a modest increase in LVEF (52.1% pre-RT to 54.3% at 5 years; *P <* 0.001) but a worsening in sensitive measures of function, such as circumferential strain (−23.7% pre-RT to −21.0% at 5 years; *P =* 0.003). Among left-sided/bilateral breast cancer participants, changes in cardiac function were observed across all parameters (*P <* 0.05). The maximum LAD dose was associated with a modest worsening in LVEF, longitudinal strain, circumferential strain, and E/e′.

**Conclusions:**

Over a median of 5.1 years, modest changes in cardiac function were observed with RT. Maximum LAD dose was associated with a worsening in systolic and diastolic function parameters.

Breast cancer treatment results in cardiac morbidity secondary to the cardiotoxic effects of systemic therapy and radiation therapy (RT).^1^^,^^2^ Specific to RT, the landmark study of women treated with breast RT between 1973 and 2001 by Darby et al^3^ demonstrated a significant relationship between the mean heart dose (MHD) and major cardiac events. More recently, advances in radiation techniques, including 3-dimensional computed tomography planning, prone positioning, and deep inspiration breath hold, have reduced incidental heart dose.^4^, ^5^, ^6^ However, the effects of modern-day RT on cardiovascular function remain incompletely understood.

While radiation-related cardiovascular toxicity has historically focused on MHD, there is growing interest in dose to cardiac substructures.^3^ Retrospective analyses suggest that RT exposure to specific regions of the heart, such as the left ventricle (LV) and left anterior descending artery (LAD), may be more indicative of cardiac risk than conventional MHD metrics.^7^, ^8^, ^9^, ^10^, ^11^, ^12^ 3-dimensional-computed tomography planning has permitted delineation of organs at risk, and artificial intelligence–derived contouring algorithms have enhanced the acquisition of the cardiac substructure dose.^13^^,^^14^

However, there remain fundamental knowledge gaps related to our understanding of RT in breast cancer and subsequent cardiovascular risk. First, the effects of modern-day RT on cardiac function remain incompletely defined. Second, the specific cardiac substructure dose metrics most indicative of potential changes in cardiac function are unknown. To address these questions, we leveraged a large longitudinal cohort of breast cancer patients receiving combination systemic cancer and RT, with detailed whole heart and substructure contouring and quantitative echocardiographic phenotyping.

## Methods

### Study population

The Cardiotoxicity of Cancer Therapy study (NCT01173341) is a longitudinal, prospective cohort study of women with breast cancer undergoing treatment with doxorubicin and/or HER2-targeted trastuzumab across multiple sites within the University of Pennsylvania Health System (NCT01173341). As previously described, women 18 years of age or older with a breast cancer diagnosis who received trastuzumab and/or doxorubicin as part of their breast cancer treatment were included.^15^^,^^16^ Systemic therapy was determined by the treating oncologist and consisted of the following 3 groups: 1) doxorubicin (240 mg/m^2^) with cyclophosphamide, followed by paclitaxel (“anthracycline”); 2) trastuzumab (with/without pertuzumab) with docetaxel and either cyclophosphamide or carboplatin (“trastuzumab”); or 3) doxorubicin (240 mg/m^2^) with cyclophosphamide, followed by trastuzumab (with/without pertuzumab) and paclitaxel (“anthracycline + trastuzumab”). Participants underwent protocol-specified, standardized echocardiograms before, during, and after breast cancer systemic therapy.

For this study, we performed a subcohort analysis of participants who received definitive adjuvant external beam RT to the breast or chest wall at the University of Pennsylvania Health System, and who had an echocardiogram performed within the 3 months prior to the start of RT (defined as “baseline”) and at least 1 follow-up echocardiogram after RT (Supplemental Figure 1). For the purposes of this analysis, the pre-RT echocardiography time point was used as the baseline. Of note, in our previously published analysis from this cohort study, baseline was noted as the start of breast cancer systemic therapy.^15^^,^^17^ This study was approved by the University of Pennsylvania Institutional Review Board. All participants provided written informed consent.

### Echocardiography acquisition and analysis

Transthoracic echocardiograms performed as part of the study protocol have previously been described in detail.^17^ Briefly, echocardiograms were acquired at an Intersocietal Accreditation Commission laboratory using Vivid 7, E9, or E95 machines (GE Healthcare). Images were archived and quantified at the University of Pennsylvania Center for Quantitative Echocardiography, using TomTec Imaging Systems software, by sonographers blinded to participant clinical characteristics and treatment parameters.

Measures of systolic and diastolic function were quantified and derived from 2-dimensional and Doppler images as previously described.^15^^,^^16^ These included left ventricular ejection fraction (LVEF), longitudinal strain, and circumferential strain, indicative of systolic function, and E/e′, indicative of diastolic function.^18^, ^19^, ^20^ Ventricular-arterial coupling (the ratio between effective arterial elastance [Ea] and end systolic elastance [Ees]) is indicative of cardiac efficiency and calculated based on previously established methods.^16^ Here, lower numbers are typically indicative of greater cardiac efficiency. Right ventricular (RV) fractional area change (FAC), derived from systolic and diastolic RV chamber areas, is a measure of global RV systolic function.

The intraobserver coefficient of variation for LVEF was 4.4%, for longitudinal strain was 10.9%, and for circumferential strain was 9.4%. The intraobserver coefficients of variation for mitral inflow and tissue Doppler velocities were 2.3% to 5.4% and for the Doppler timing intervals used to derive Ees were 0.3% to 5.7%. The intraobserver coefficient of variation was 5.2% for RV FAC.

### Radiation delivery and cardiac dose metrics

All participants underwent planning with 3-dimensional-computed tomography simulation. Techniques including deep inspiration breath hold, prone positioning, 3-dimensional conformal RT, intensity-modulated RT, and/or proton RT were implemented at the discretion of the treating physician. Clinical target volumes were contoured by the treating physician in accordance with standard protocol (breast/chest wall, with/without nodal volumes).^21^ RT was planned using the Eclipse treatment planning system (version 11.0-16.0, Varian Medical Systems).

To derive the RT dose to the whole heart and cardiac substructures, TheraPanacea 2.1 software was used to auto-segment the whole heart, atria, ventricles, and LAD. This validated, clinically acceptable software is based on established principles from the 2017 cardiac contouring atlas.^22^^,^^23^ Each auto-segmented contour was manually reviewed and edited as needed by 2 physicians (E.B. and C.H.). The dose metrics were calculated based on these contours (Supplemental Methods).

### Statistical methods

Baseline characteristics and cardiac dose metrics were summarized using median (Q1-Q3) for continuous variables and count and percentage for categorical variables. Normality was assessed using the Shapiro-Wilk test and Q-Q plots. The main analyses were stratified by treatment site (right vs bilateral/left). In secondary analyses, we also stratified patients by systemic cancer therapy regimen (anthracycline, trastuzumab, or anthracycline + trastuzumab). The Wilcoxon rank sum test was used to identify differences in RT dose metrics between treatment sites. A *P* value <0.05 was considered statistically significant.

The predicted mean change in echocardiographic parameters (LVEF, longitudinal strain, circumferential strain, E/e′, Ea/Ees, RV FAC) over time from baseline (pre-RT) and at 0.5, 1, 2, 3, 4, and 5 years was estimated by repeated-measures linear regression via generalized estimating equations to focus on population-averaged effects. We constructed generalized estimating equation models using an identity link and an independent working correlation structure. A priori, we defined the following baseline covariates as confounders, based on clinical judgment: time since initiation of radiation, time since chemotherapy exposure to radiation start date, systemic cancer therapy, age, cancer stage (1 and 2 vs 3 and 4), body mass index, smoking status, systolic blood pressure, race, hypertension, diabetes, cardiovascular medications (beta-blockers, angiotensin-converting enzyme inhibitors, angiotensin receptor blockers, statins), and the echocardiographic measure of interest. In models across all participants, treatment site (left/bilateral vs right) was also included as a covariate. The time since initiation of radiation was nonparametrically incorporated using cubic splines with 3 degrees of freedom to account for the potential nonlinear effects of time. A robust variance estimator was applied to account for any clustering within subjects. The results of the generalized estimating equation models are presented as the predicted mean outcome, calculated from the least-squares mean of outcomes at each level of “years from baseline,” along with the 95% CI for the predicted mean outcome and the *P* value for “years from baseline.”

For models estimating the association between cardiac radiation dose metrics and echocardiographic parameters, we applied the same modeling approach and included the same clinical covariates in the multivariable adjustment. We modeled associations across “all participants” and also stratified these analyses by “bilateral/left” or “right treatment site.” For the “all participants” analysis, we adjusted for treatment site. In these models, the primary exposure was each specific radiation dose metric (MHD, LAD maximum dose, mean LV dose, mean RV dose), and the outcomes were defined as the changes in echocardiographic parameters from baseline. To compare the strength of the associations across models, radiation dose metrics were standardized by calculating the difference between each dose metric parameter and its median, and then dividing by its interquartile range specific to each treatment site. Interquartile range was preferred over standard deviation due to the skewness of the data. The results of the generalized estimating equation models are presented as the coefficient estimates β for the standardized radiation dose metric, along with the 95% CI and *P* value for the corresponding metric.

We performed several exploratory analyses. First, we evaluated the changes in echocardiographic parameters (mean [95% CI]) over time, stratified by systemic cancer therapy (anthracycline, trastuzumab, anthracycline + trastuzumab) exposure. We then explored the associations between radiation dose volume metrics and echocardiographic parameters, stratified by systemic cancer therapy exposure. Moreover, we also estimated the mean (95% CI) echocardiographic parameter over time according to the German Society for Radiation Oncology heart guidelines, defined as mean LAD dose </≥10 Gy, mean LV dose </≥3 Gy, and LV V5 </≥17%.

All analyses were performed by R 4.3.1 (R Foundation for Statistical Computing).

## Results

### Study population

There were 303 participants who received RT for their breast cancer from 2010 to 2019, with a median of 7 echocardiograms per participant over a median follow-up time of 5.1 years (Q1-Q3: 3.2-7.1 years). Table 1 details the clinical characteristics for the cohort. The median age was 52 years (Q1-Q3: 44-60 years). Approximately 61.7% of participants were White, 33.3% were African American, and 3.0% were Asian. Cardiovascular risk factors were highly prevalent, with hypertension (33.0%), hyperlipidemia (22.1%), diabetes mellitus (9.9%), and current or former smoking history (36.0%) being common.

**Table 1:** Baseline Characteristics of Study Participants by Treatment Site

	All Participants (N = 303)	Bilateral or Left Breast or Chest Wall (n = 164)	Right Breast or Chest Wall Only (n = 139)
Age, y	52 (44-60)	51 (42-59)	52 (45-61)
Race			
African American	101 (33.3)	54 (32.9)	47 (33.8)
White	187 (61.7)	102 (62.1)	85 (61.2)
Asian	9 (3.0)	8 (4.9)	1 (0.7)
Ethnicity			
Hispanic/Latino	5 (1.7)	0 (0)	5 (3.6)
Not Hispanic/Latino	297 (98.0)	164 (100)	133 (95.7)
AJCC breast cancer stage			
1	58 (19.1)	30 (18.3)	28 (20.1)
2	152 (50.2)	85 (51.8)	67 (48.2)
3	91 (30.0)	47 (28.7)	44 (31.7)
4	2 (0.7)	2 (1.2)	0 (0)
Systemic cancer therapy			
Anthracycline	187 (61.7)	105 (64.0)	82 (59.0)
Anthracycline + trastuzumab	51 (16.8)	27 (16.5)	24 (17.3)
Trastuzumab	65 (21.5)	32 (19.5)	33 (23.7)
BMI, kg/m^2^	28 (24-32)	27 (24-32)	28 (24-33)
SBP, mm Hg	122 (113-133)	122 (113-134)	122 (114-132)
DBP, mm Hg	75 (69-81)	75 (69-82)	75 (69-81)
Tobacco use^a^			
Current or former	109 (36.0)	62 (37.8)	47 (33.8)
Never	193 (63.7)	101 (61.6)	92 (66.2)
Diabetes	30 (9.9)	19 (11.6)	11 (7.9)
Cardiovascular risk factors/disease			
Hypertension	100 (33.0)	54 (32.9)	46 (33.1)
Hyperlipidemia	67 (22.1)	38 (23.2)	29 (20.9)
MI/CAD	6 (2.0)	5 (3.1)	1 (0.7)
Heart failure	22 (7.3)	12 (7.3)	10 (7.2)
Arrhythmia	8 (2.7)	3 (1.8)	5 (3.6)
Cardiovascular medications			
Beta-blocker	34 (11.2)	20 (12.2)	14 (10.1)
Statins	39 (12.9)	20 (12.2)	19 (13.7)
ACE inhibitor/ARB	55 (18.2)	27 (16.5)	28 (20.1)
Diuretic	46 (15.2)	21 (12.8)	25 (18.0)

Values are median (Q1-Q3) or n (%).

ACE = angiotensin-converting enzyme; AJCC = American Joint Committee on Cancer; ARB = angiotensin receptor blocker; BMI = body mass index; CAD = coronary artery disease; DBP = diastolic blood pressure; E = early diastolic mitral inflow velocity; e′ = early diastolic mitral annular tissue velocity; MI = myocardial infarction; SBP = systolic blood pressure.

a There was 1 participant with unknown tobacco use history.

Most participants had American Joint Committee on Cancer 7th edition stage 2 (50.2%) or 3 (30.0%) breast cancer, followed by stage 1 breast cancer (19.1%). Anthracycline without trastuzumab was administered in 61.7%, trastuzumab without anthracycline in 21.5%, and anthracycline + trastuzumab in 16.8%. RT site laterality was balanced, with 156 (51.5%) participants treated to the left breast/chest wall, 139 (45.9%) participants treated to the right breast/chest wall, and 8 (2.6%) participants treated bilaterally. Across all participants, the median total RT dose was 52.6 Gy (Q1-Q3: 50.4-60.0 Gy) and the median total number of fractions was 28 (Q1-Q3: 21-30). The majority of plans utilized photons (95.0%). Deep inspiration breath hold was used in 58 (19.1%) participants, mostly those with left or bilateral breast/chest wall cancer (n = 57 of 58 [98.3%]). Prone positioning was implemented in 24 (7.9%) participants, again, primarily in left-sided breast cancer (19 [79.2%] of 24 prone treatments).

### Cardiac radiation dose metrics

We derived cardiac radiation dose metrics for the whole heart, LAD, LV, and RV for all participants (Table 2). The overall median MHD was 1.19 Gy (Q1-Q3: 0.75-2.61 Gy). For all cardiac structures and dose metrics, there was a significant difference between the RT treatment site groups, bilateral/left and right (*P <* 0.001). The median MHD was approximately 3 times higher for bilateral/left group (2.30 Gy [Q1-Q3: 1.45-3.49 Gy]) than for the right group (0.76 Gy [Q1-Q3: 0.52-0.97]). The differences between left/bilateral and right treatment groups were most pronounced for the dose to the LAD. The median LAD maximum dose for the left/bilateral group was 39.78 Gy (Q1-Q3: 22.81-46.94 Gy) and the for the right group was 0.45 (Q1-Q3: 0.27-0.68).

**Table 2:** Radiation Treatment Characteristics and Cardiac Dose Metrics by Treatment Site

	All Participants (N = 303)	Bilateral or Left Treatment Site (n = 164)	Right Treatment Site (n = 139)
Radiation treatment characteristic			
Radiation type			
Photon	288 (95.0)	157 (95.7)	131 (94.2)
Proton	15 (5.0)	7 (4.3)	8 (5.8)
Breast/chest wall irradiation sites			
Bilateral	8 (2.6)	8 (4.9)	0 (0)
Left	156 (51.5)	156 (95.1)	0 (0)
Right	139 (45.9)	0 (0)	139 (100)
Treatment of IMNs	102 (33.6)	54 (32.9)	48 (34.5)
Treatment of supraclavicular nodes	173 (57.1)	96 (58.5)	77 (55.4)
Deep inspiration breath hold	58 (19.1)	57 (34.8)	1 (0.7)
Prone positioning	24 (7.9)	19 (11.6)	5 (3.6)
Cardiac radiation dose metrics			
Heart			
Mean, Gy	1.19 (0.75-2.61)	2.30 (1.45-3.49)	0.76 (0.52-0.97)
Maximum, Gy	26.62 (5.71-46.63)	44.82 (37.31-49.09)	5.67 (4.29-8.19)
V5 Gy, %	2.2 (0.1-8.4)	6.7 (3.0-12.5)	0.1 (0.0-1.1)
V20 Gy, %	0.0 (0.00-1.8)	1.4 (0.3-3.7)	0.0 (0.0-0.0)
LAD			
Mean, Gy	3.02 (0.20-13.12)	12.04 (5.41-18.85)	0.20 (0.09-0.32)
Maximum, Gy	8.91 (0.48-40.96)	39.78 (22.81-46.94)	0.45 (0.27-0.68)
V5 Gy, %	8.0 (0.0-64.0)	60.8 (37.2-76.8)	0.0 (0.0-0.0)
V20 Gy, %	0.0 (0.0-23.1)	19.3 (0.4-40.3)	0.0 (0.0-0.0)
Left ventricle			
Mean, Gy	1.10 (0.19-3.11)	2.84 (1.68-4.61)	0.19 (0.09-0.26)
Maximum, Gy	5.71 (0.63-39.24)	37.72 (18.97-47.05)	0.62 (0.39-0.79)
V5 Gy, %	0.2 (0.0-10.7)	9.2 (2.3-19.5)	0.0 (0.0-0.0)
V20 Gy, %	0.0 (0.0-1.2)	1.1 (0.0-4.4)	0.0 (0.0-0.0)
Right ventricle			
Mean, Gy	1.13 (0.58-2.21)	2.04 (1.33-3.02)	0.60 (0.37-0.86)
Maximum, Gy	4.40 (2.02-18.37)	11.88 (5.72-34.89)	2.00 (1.29-2.74)
V5 Gy, %	0.0 (0.0-4.6)	3.0 (0.2-11.3)	0.0 (0.0-0.0)
V20 Gy, %	0.0 (0.0-0.0)	0.0 (0.0-1.1)	0.0 (0.0-0.0)

Values are n (%) or median (Q1-Q3).

IMN = internal mammary node; LAD = left anterior descending artery.

### Longitudinal changes in echocardiographic parameters of systolic and diastolic cardiac function

**Figure 1:**
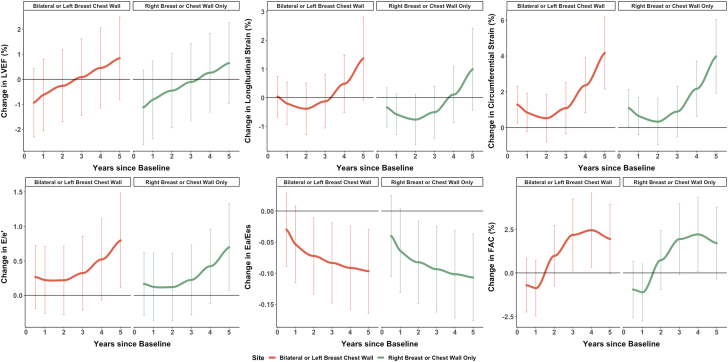
Change in Echocardiogram Parameters Over Time, by Treatment Site Plots are stratified by treatment sites bilateral or left chest wall (orange) and right breast or chest wall only (green) and correspond to the predicted mean change in echocardiogram parameter over time from baseline (time = 0, pre–radiation therapy [pre-RT]) and at 0.5, 1, 2, 3, 4, and 5 years and were estimated by repeated-measures linear regression via generalized estimating equations. The model was adjusted for the following covariates: age, cancer stage (1 and 2 vs 3 and 4), body mass index, smoking status, systolic blood pressure, race, baseline hypertension, baseline diabetes, baseline cardioprotective medications (beta-blockers, angiotensin-converting enzyme inhibitors, angiotensin receptor blockers, statins), time since last chemotherapy exposure to start of radiation, systemic cancer therapy (anthracycline, trastuzumab, anthracycline + trastuzumab), treatment site (left/bilateral, right), time since initiation of radiation, and baseline echocardiographic measure of interest. Lines within bars represent 95% CIs. E = peak early diastolic transmitral blood flow; e′ = peak early mitral annular tissue velocity; Ea = systemic arterial load; Ees = left ventricular contractile function; FAC = fractional area change; LVEF = left ventricular ejection fraction.

The changes in echocardiographic measures of systolic and diastolic cardiac function with radiation exposure were then evaluated using the generalized estimating equations model described in the Methods (Central Illustration, Figure 1, Supplemental Tables 1A to 1C). We observed a very modest, statistically but likely less clinically significant increase in LVEF over time, with a pre-RT LVEF of 52.1% to 54.3% at 5 years (*P <* 0.001) (Supplemental Table 1A). Of note, the baseline pre-RT echocardiogram values reported represent those at the start of RT, after completion of chemotherapy. These baseline pre-RT values were lower than that prechemotherapy; for example, LVEF and longitudinal strain prechemotherapy were 57.0% and −17.1%, respectively.

**Central Illustration:**
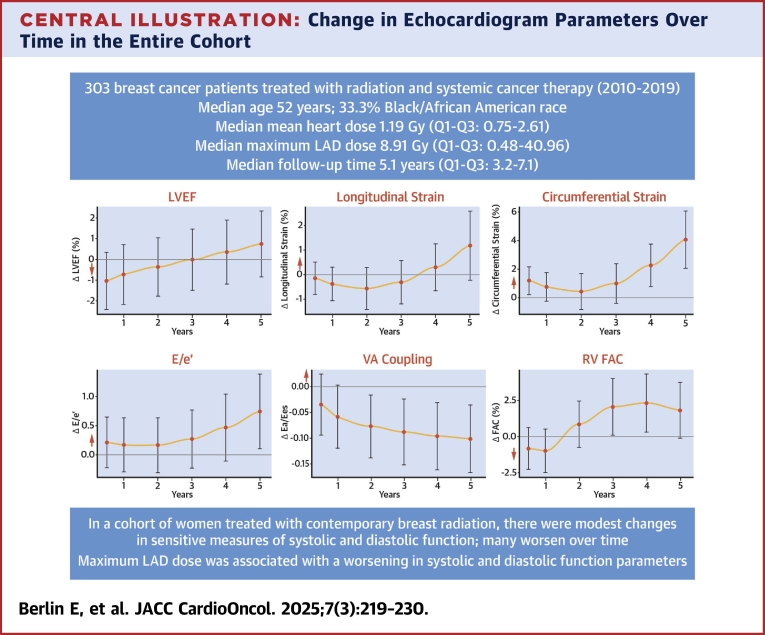
Change in Echocardiogram Parameters Over Time in the Entire Cohort Plots correspond to the predicted mean change in echocardiogram parameter over time from baseline (time = 0, pre–radiation therapy [pre-RT]) and at 0.5, 1, 2, 3, 4, and 5 years and were estimated by repeated-measures linear regression via generalized estimating equations. The model was adjusted for the following covariates: age, cancer stage (1 and 2 vs 3 and 4), body mass index, smoking status, systolic blood pressure, race, baseline hypertension, baseline diabetes, baseline cardioprotective medications (beta-blockers, angiotensin-converting enzyme inhibitors, angiotensin receptor blockers, statins), time since last chemotherapy exposure to start of radiation, systemic cancer therapy (anthracycline, trastuzumab, anthracycline + trastuzumab), time since initiation of radiation, treatment site (left/bilateral vs right), and baseline echocardiographic measure of interest. Lines within bars represent 95% CIs. Along the y-axis, a bold red arrow is placed to indicate the directionality of a detriment in cardiovascular function relative to baseline. The x-axis represents the years since radiation therapy initiation. E = early diastolic mitral inflow velocity; e′ = early diastolic mitral annular tissue velocity; Ea = systemic arterial load; Ees = left ventricular contractile function; FAC = fractional area change; LVEF = left ventricular ejection fraction; RT = radiation therapy; VA = ventricular-arterial.

Similarly, measures of ventricular-arterial coupling (Ea/Ees) showed a modest, statistically significant decrease over time, from 1.02 pre-RT to 0.92 at 5 years (*P <* 0.001). RV FAC also improved over time (47.4% pre-RT to 48.2% at 5 years; *P =* 0.004). However, there was a statistically significant worsening of circumferential strain (−23.7% pre-RT to −21.0% at 5 years; *P =* 0.003). Among those with left-sided/breast cancer, these findings were all statistically significant. Sensitive measures of cardiac function, most notably longitudinal and circumferential strain (*P <* 0.001) and diastolic function, as defined by E/e′ (*P =* 0.045), all worsened over time (Figure 1, Supplemental Table 1B).

In exploratory analysis stratified by systemic cancer therapy (Supplemental Tables 2A to 2D), we identified modest differences in MHD, with trastuzumab patients receiving a lower mean heart dose of 0.98, compared with 1.22 and 1.28 in the anthracycline and anthracycline + trastuzumab subgroups (*P =* 0.040). Consistent with this, there was no significant worsening in sensitive measures of cardiac function (longitudinal, circumferential strain) over time on population average in the trastuzumab subgroup, but there was a modest worsening in these measures in the anthracycline chemotherapy subgroups (Supplemental Tables 2A to 2D, Supplemental Figure 2).

### Associations between cardiac function and radiation dose metrics

We then determined the potential functional importance of each radiation dose metric parameter, as defined by the strength of the association between the radiation dose metric and echocardiographic parameter (Table 3). Most notable, the maximum LAD dose was associated with a multiple echocardiographic parameters indicative of global function. For each interquartile range increase in maximum LAD dose, there was a 1.1% (95% CI: 0.2% to 1.9%) worsening in longitudinal strain and a 2.1% (95% CI: −3.4% to −0.8%) worsening in LVEF as well as a worsening in diastolic function as represented by E/e′ (0.5; 95% CI: 0.0-0.9) across all participants. In stratified analysis by bilateral/left treatment site, there was also an association between maximum LAD dose and circumferential strain (0.6; 95% CI: 0.0-1.3).

**Table 3:** Association Between Radiation Dose Metrics and Cardiac Function Parameters Across All Time Points: MHD, LAD Maximum Dose, Mean LV Dose, and Mean RV Dose

	All Participants		Bilateral or Left Treatment Site		Right Treatment Site	
	β (95% CI)	*P* Value	β (95% CI)	*P* Value	β (95% CI)	*P* Value
MHD						
LVEF, %	−0.39 (−1.12 to 0.34)	0.29	0.03 (−0.59 to 0.65)	0.93	−1.00 (−1.26 −0.75)	<0.001
Longitudinal strain, %	0.29 (−0.03 to 0.61)	0.079	0.20 (−0.19 to 0.58)	0.33	0.31 (0.19–0.42)	<0.001
Circumferential strain, %	0.03 (−0.28 to 0.34)	0.84	0.09 (−0.39 to 0.57)	0.72	0.02 (−0.25 to 0.29)	0.86
E/e′	0.13 (−0.05 to 0.32)	0.15	0.18 (−0.01 to 0.38)	0.069	−0.01 (−0.12 to 0.11)	0.93
Ea/Ees	0.01 (−0.03 to 0.04)	0.72	0.00 (−0.04 to 0.03)	0.91	0.02 (−0.00 to 0.05)	0.064
RV FAC, %	0.20 (−0.51 to 0.90)	0.59	0.20 (−0.71 to 1.10)	0.67	−0.04 (−0.82 to 0.74)	0.92
LAD maximum dose						
LVEF, %	−2.12 (−3.44 to −0.80)	0.002	−1.01 (−1.74 to −0.28)	0.007	−0.03 (−0.07 to 0.01)	0.090
Longitudinal strain, %	1.05 (0.24–1.86)	0.011	0.56 (0.11–1.00)	0.015	0.19 (0.11–0.28)	<0.001
Circumferential strain, %	1.00 (−0.18 to 2.18)	0.098	0.63 (0.01–1.26)	0.048	0.00 (−0.08 to 0.08)	0.99
E/e′	0.45 (−0.01 to 0.91)	0.052	0.20 (−0.06 to 0.46)	0.12	−0.01 (−0.02 to 0.00)	0.020
Ea/Ees	0.04 (−0.02 to 0.10)	0.19	0.03 (−0.01 to 0.06)	0.14	0.00 (−0.00 to 0.00)	0.45
RV FAC, %	0.54 (−2.03 to 3.11)	0.68	0.24 (−1.21 to 1.69)	0.75	−0.02 (−0.05 to 0.01)	0.21
Mean LV Dose						
LVEF, %	−0.43 (−1.13 to 0.27)	0.22	−0.34 (−1.08 to 0.40)	0.37	−0.89 (−1.64 to −0.15)	0.019
Longitudinal strain, %	0.34 (−0.06 to 0.74)	0.095	0.30 (−0.10 to 0.70)	0.15	0.50 (0.23–0.78)	<0.001
Circumferential strain, %	0.12 (−0.37 to 0.60)	0.64	0.19 (−0.41 to 0.79)	0.53	−0.07 (−0.44 to 0.31)	0.72
E/e′	0.18 (−0.06 to 0.42)	0.14	0.20 (−0.03 to 0.43)	0.083	0.02 (−0.17 to 0.21)	0.84
Ea/Ees	0.01 (−0.03 to 0.05)	0.73	0.01 (−0.04 to 0.05)	0.80	0.02 (0.00–0.04)	0.053
RV FAC, %	0.16 (−0.80 to 1.12)	0.74	0.04 (−1.05 to 1.13)	0.94	0.28 (−0.41 to 0.97)	0.42
Mean RV dose						
LVEF, %	−0.03 (−0.40 to 0.34)	0.88	0.21 (−0.03 to 0.45)	0.084	−0.54 (−0.65 to −0.43)	<0.001
Longitudinal strain, %	0.10 (−0.06 to 0.27)	0.23	0.05 (−0.16 to 0.26)	0.62	0.17 (0.11–0.22)	<0.001
Circumferential strain, %	−0.02 (−0.13 to 0.09)	0.77	−0.02 (−0.20 to 0.17)	0.86	0.00 (−0.12 to 0.12)	0.95
E/e′	0.04 (−0.05 to 0.13)	0.41	0.06 (−0.04 to 0.15)	0.23	0.02 (−0.02 to 0.05)	0.35
Ea/Ees	0.00 (−0.02 to 0.01)	0.54	−0.01 (−0.02 to 0.01)	0.21	0.01 (0.00–0.02)	0.079
RV FAC, %	0.15 (−0.10 to 0.41)	0.24	0.18 (−0.17 to 0.54)	0.32	−0.01 (−0.84 to 0.82)	0.98

A generalized estimating equations model was adjusted for the following covariates: age, cancer stage (1 and 2 vs 3 and 4), body mass index, smoking status, systolic blood pressure, race, baseline hypertension, baseline diabetes, baseline cardioprotective medications (beta-blockers, angiotensin-converting enzyme inhibitors, angiotensin receptor blockers, statins), time since last chemotherapy exposure to start of radiation, time since initiation of radiation, systemic cancer therapy (anthracycline, trastuzumab, anthracycline + trastuzumab), and baseline echocardiographic measure of interest. Site was additionally adjusted for the model including all participants. β indicates thecoefficient estimates for the interquartile range standardized mean RV dose.

Ea = systemic arterial load; Ees = left ventricular contractile function; FAC = fractional area change; LVEF = left ventricular ejection fraction; MHD = mean heart dose; RV = right ventricular; other abbreviations as in Table 1.

Although we did not determine a consistent, statistically significant interaction by treatment group (anthracycline, trastuzumab, anthracycline + trastuzumab) and radiation dose volume metric (MHD, LAD maximum dose, mean LV dose, mean RV dose) (*P* > 0.05, data not shown), we performed exploratory analysis stratified by systemic cancer therapy. There were significant associations between each of the radiation dose metrics and measures of systolic and diastolic function in those receiving anthracycline chemotherapy, either with or without trastuzumab, and some associations between maximum LAD dose and mean LV dose and cardiac function in the trastuzumab subgroup (Supplemental Tables 3A to 3D).

In an exploratory analysis using the German Society for Radiation Oncology criteria, there were significant differences in longitudinal strain (*P =* 0.027) and Ea/Ees (*P =* 0.042) according to LAD dose </≥10 Gy, and for circumferential strain (*P =* 0.049) and Ea/Ees (*P =* 0.024) for mean LV dose </≥3 Gy, with higher doses indicative of worse function (Supplemental Tables 4A to 4C). There were no significant differences in echocardiographic measure for LV V5 </≥17%.

## Discussion

To our knowledge, this is the largest longitudinal prospective cohort study in modern-day breast RT with detailed echocardiographic phenotyping and radiation dose metrics. There are 3 principal findings from our analysis. First, modern breast RT, in conjunction with systemic cancer therapy, results in a modest worsening in measures of longitudinal and circumferential strain and diastolic function, but not LVEF, most notably in those receiving left-sided/bilateral radiation, over a median follow-up of 5.1 years. We believe this is related to the dose delivered and potentially indicative of an early heart failure with preserved ejection fraction (HFpEF) phenotype. Second, increasing radiation dose to the heart and cardiac substructures is associated with a modest worsening in systolic and diastolic function. Third, in comparing the various radiation dose metrics, increases in the maximum dose to the LAD are associated with a greater number of measures of global systolic and diastolic function, suggestive of the importance of radiation dose to the LAD.

Our prior work in a much smaller cohort of breast cancer participants also suggested very minor changes in LVEF with RT that were of borderline statistical significance and not clinically significant.^24^ Similar findings were also shown in work by Yu et al,^25^ in which in 47 HER2+ breast cancer patients receiving anthracyclines and trastuzumab there was no association between MHD and changes in LVEF, strain, and diastolic indices 6 months after RT. Our current study substantially expands on the existing literature through a considerably larger sample size, longer follow-up, and more comprehensive echocardiographic and RT dose phenotyping. We believe that this study provides clear evidence that contemporary breast RT on population average does not result in significant, clinically overt cardiac systolic dysfunction in the first few years following RT. The statistically significant changes in systolic function parameters of LVEF during the study period were modest (eg, LVEF from 52.1% to 54.3%, circumferential strain from −23.7% to −21.0%) and worsened over time (eg, greater in years 4-5), and we postulate were potentially related to the early stabilization of cardiac function after exposure to cardiotoxic systemic cancer therapy with anthracyclines with or without trastuzumab, and were potentially indicative of a developing HFpEF phenotype (E/e′ from 8.6 to 9.1).

We further propose that the low heart dose with modern-day RT is largely responsible for our observations. Significant improvements in RT dose delivery have been made in the past 2 decades. The implementation of computed tomography planning in the early 2000s allowed for 3-dimensional visualization of the heart and other organs at risk and more accurate measurement of cardiac radiation exposure.^26^^,^^27^ This, in turn, has made it possible for optimization of organs at risk sparing with 3-dimensional-computed tomography–informed adjustment of radiation beams and patient positioning. Techniques such as deep inspiration breath hold and prone positioning, which were adopted in the early 2010s, have created more strategies to reduce heart dose, by increasing the distance between the radiation target and the heart.^28^, ^29^, ^30^ The low MHD (1.19 Gy overall and 2.30 Gy in left/bilateral breast cancer) in our cohort reflects these advancements, significantly lower from the historical average MHD of about 5 Gy.^3^ Moreover, our findings of significant associations in left-sided/bilateral participants, and not in right-sided participants, support this.

In breast cancer, the RT target directly abuts the anterior portion of the heart; thus, ongoing science is focused on defining the functional and clinical significance of the dose to anterior cardiac structures. Recent investigations suggest that dose to the LV and LAD are associated with cardiac effects. For example, the BACCARAT (BreAst Cancer and CArdiotoxicity Induced by RAdioTherapy) trial found an association between the LV V5 and subclinical LV dysfunction at 6 months post-RT, and in a subsequent analysis found a dose-response relationship between LV exposure and coronary artery calcification.^31^^,^^32^ The Breast Cancer Working Group of German Society for Radiation Oncology proposed dose constraints for cardiac substructures, including a mean LV dose of <3 Gy, LV V5 of <17%, and a mean LAD dose of <10 Gy,^33^ and our exploratory analysis determined some significant differences in terms of the trajectories of cardiac function according to these cutpoints.

However, cardiac substructure constraints have not been routinely adopted in clinical radiation oncology practice. This may be due to practical considerations, including the inherent challenges of contouring these regions. While auto-segmentation software has improved the consistency and efficiency of generating cardiac substructure contours, these contours still require manual review. In addition, the LAD and other coronary vasculature are challenging to visualize on noncontrast RT planning scans. As we determined that dose to the LAD was associated with several measures of cardiac function, our findings suggest a need for advances in the accurate and precise quantitation and eventual implementation of routine evaluation of cardiac substructure dose.

While overall we did not observe any clinically overt and substantial worsening in cardiac systolic function over time, we did determine that increases in cardiac RT dose were associated with echocardiographic measures of diastolic function (E/e′) and sensitive measures of systolic function (circumferential strain, longitudinal strain, ventricular-arterial coupling), particularly notable across bilateral/left-sided participants, and particularly worse over time (greater in years 4-5). These associations support our premise that detailed evaluation of echocardiographic time points immediately prior to and after RT provides insights into the cardiac effects of RT, and demonstrate that cardiac exposure to RT has a dose-dependent, detrimental effect on cardiac function, further emphasizing the importance of minimizing heart dose. We believe that these findings also provide functional data to support that the primary clinical heart failure phenotype observed with RT is HFpEF. In a population-based case-control study by Saiki et al^34^ of 170 women treated with breast radiation from 1998 to 2013 who were diagnosed with heart failure, the majority (64%) had HFpEF. The relative risk of developing HFpEF increased with MHD, whereby the OR for HFpEF per log MHD was 16.9 (*P <* 0.001) and remained significant after adjustment for heart failure risk factors and cancer stage (OR: 22.7; *P <* 0.001). Greater awareness of this potential clinical phenotype is needed in the oncology and cardiology communities.

Among the cardiac structures studied in our analysis, the dose to the LAD demonstrated several statistically significant associations with measures of global cardiac function. In the existing literature, radiation dose to the LAD is typically associated with coronary artery stenosis, perfusion deficits, and ischemic events.^7^^,^^32^^,^^35^ Our findings suggest that LAD irradiation can also affect global cardiac function, and that perhaps the LAD maximum dose is a more sensitive dose metric than MHD. Interestingly, HFpEF has been linked to coronary vascular impairment, which may offer a potential mechanistic basis for our observations.^36^^,^^37^

We believe our findings have important clinical implications for counseling women undergoing RT for their breast cancer, many of whom are concerned about potential cardiac effects. Our findings provide reassurance as to the lack of clinically overt, detrimental changes in cardiac systolic function over a median follow-up of 5.1 years following RT for breast cancer, likely secondary to low heart dose. Our results also suggest that changes in diastolic function are observed in the immediate time period following RT, and that over the longer term (as this overt phenotype may take years to develop), clinicians need to be aware of the risk of HFpEF, particularly in individuals who receive higher heart dose. Finally, our data also support the importance of the dose to the LAD and motivate additional science to improve contouring of the LAD and associated substructures.

### Study limitations

All participants received systemic cancer therapy with possible cardiotoxic effects; thus, we cannot completely divorce our findings from the potential confounding effects of anthracyclines and/or trastuzumab. However, we did fully adjust for systemic therapy in our statistical analysis, and these are highly relevant treatment paradigms that enhance the generalizability of our findings to patients cared for in everyday clinical practice in the modern treatment era. We additionally explored stratified analyses by systemic cancer therapy. While we observed worse changes in cardiac function in those receiving anthracycline containing regimen, lending support to the “two-hit” hypothesis, our findings further emphasize that even in conjunction with potentially cardiotoxic systemic cancer therapy we did not observe clinically substantial cardiac dysfunction with RT in the intermediate term on population average.^38^^,^^39^ Moreover, our observed associations between radiation dose volume metrics and cardiac function support our approach in evaluating timepoints immediately prior to and after RT. However, we fully acknowledge that longer follow-up is also needed to determine the impact on clinical endpoints and, importantly, coronary artery disease, as phenotypes such as HFpEF and coronary stenosis may take years to develop. Over a median follow-up of 5.1 years, incident cardiac dysfunction was low, with LV systolic dysfunction (LVEF decrease ≥10% to <50%) at 5.9% and diastolic dysfunction (E/e′ >14) at 8.9%. We also did not include a non-RT subgroup in this analysis. We also note important strengths of our study, including the cohort size, racial diversity, detailed cardiac segmentation, quantitative echocardiography, comprehensive longitudinal follow-up, and the clinical relevance to modern-day RT.

## Conclusions

In a large prospective cohort of women undergoing modern, multimodality breast cancer treatment with detailed echocardiographic assessment and contouring of RT dose metrics, we found that generally low cardiac dose exposure did not result in detrimental effects on LVEF over a median follow-up of 5.1 years. The changes in echocardiographic measures of cardiac function during the first 5 years after RT are most indicative of worsening diastolic function, and circumferential and longitudinal strain. We believe that these changes, coupled with a decrease in ventricular-arterial coupling, suggest an early heart failure with preserved ejection fraction phenotype. Our data also suggest that it remains important to continue efforts to minimize heart dose, given that incremental increases in radiation dose to the heart and substructures (LAD) are associated with changes in cardiac function.

Perspectives **COMPETENCY IN MEDICAL KNOWLEDGE:** The effects of modern breast RT, in conjunction with systemic cancer therapy, result in modest changes in diastolic function and measures of circumferential and longitudinal strain. Increasing the radiation dose to the heart and cardiac substructures is associated with a modest worsening in these indices and a decrease in ventricular-arterial coupling. Increases in the maximum dose to the LAD are associated with multiple measures of global systolic and diastolic function, suggestive of the importance of radiation dose to the LAD.

**TRANSLATIONAL OUTLOOK:** Our findings provide reassurance as to the lack of overt, clinically substantial detrimental changes in LVEF in the intermediate term following RT for breast cancer, likely secondary to the low heart dose. Our results also suggest that changes in diastolic function, strain, and ventricular-arterial coupling are observed following RT, potentially providing early indication of the subsequent risk of HFpEF. Finally, our data also support the importance of dose to the LAD and motivate additional science to improve contouring of the LAD and associated substructures.

## Funding Support and Author Disclosures

This work was supported by funding and support from the National Institutes of Health (grant numbers R01HL118018, R21 HL-157886, and K24HL167127-01A1 to Dr Ky); and the American Heart Association AHA Strategically Focused Research Network Award in Cardio-Oncology to Dr Ky, and Abramson Cancer Center Pilot Grant to Drs Ky and Freedman. Dr Taunk has received grant support from Varian Medical Systems and TheraPanacea; served as a consultant for Boston Scientific and Point Biopharma; received honoraria from GenMab and Boston Scientific; and served on the advisory board for Boston Scientific, Point Biopharma, and Varian Medical Systems. Dr Narayan has served as a consultant for Johnson & Johnson, Pfizer, Regeneron, Astellas, Merck, Xencor, Myovant, Sanofi, Exelixis, and Eisai; and received institutional research funding from Pfizer, Merck, Johnson & Johnson, Bristol Myers Squibb, Regeneron, and Xencor. Dr Clark has received grant support from Lilly. Dr Shah has served as a consultant for Gilead Sciences, Daiichi-Sankyo, and Biotheranostics. Dr Ky has received grant support from Pfizer; honoraria from *UpToDate* and the American College of Cardiology; and has provided service as the echo core lab (no direct compensation) for Impulse Dynamics. All other authors have reported that they have no relationships relevant to the contents of this paper to disclose.
