# Supplementary material for: Cardiac Effects of Modern Breast Radiation Therapy in Patients Receiving Systemic Cancer Therapy
Source: JACC CardioOncol. 2025 Mar 4;7(3):219–30. doi: 10.1016/j.jaccao.2025.01.012 (PMC12046838; doi:10.1016/j.jaccao.2025.01.012)
Supplement: Supplemental Material [file mmc1.docx]

**Cardiac Effects of Modern Breast Radiation Therapy in Patients Receiving Systemic Cancer Therapy Supplemental File**

**Supplemental Methods: Cardiac Dose Metrics**

A plan sum was created for each participant to reflect the delivered plans and dose actually delivered based on treatment record. When dose accumulation was needed across multiple scans, image fusion was prioritized in the heart area to keep an accurate heart dose accumulation. The dose metrics for the whole heart, LV, RV, and LAD were extracted from the plan sum. Dose metrics for these substructures included the mean doses and maximum doses. Maximum doses correspond to the highest dose to 0.03 cm^3^ of a given structure (D_0.03cc_).

**Supplemental Figure 1: Echocardiography Protocol**

This study is a subcohort analysis of breast cancer participants (NCT01173341) who received external beam radiation therapy (RT), focusing specifically on the echocardiogram (echo) acquisition timepoints prior to radiation therapy and at 6 months, 1 year, 2 years 3, 4 and 5 years after RT. Non-HER2 systemic therapy was completed prior to RT start, whereas HER2 therapy was delivered both prior to, during, and after RT.

**
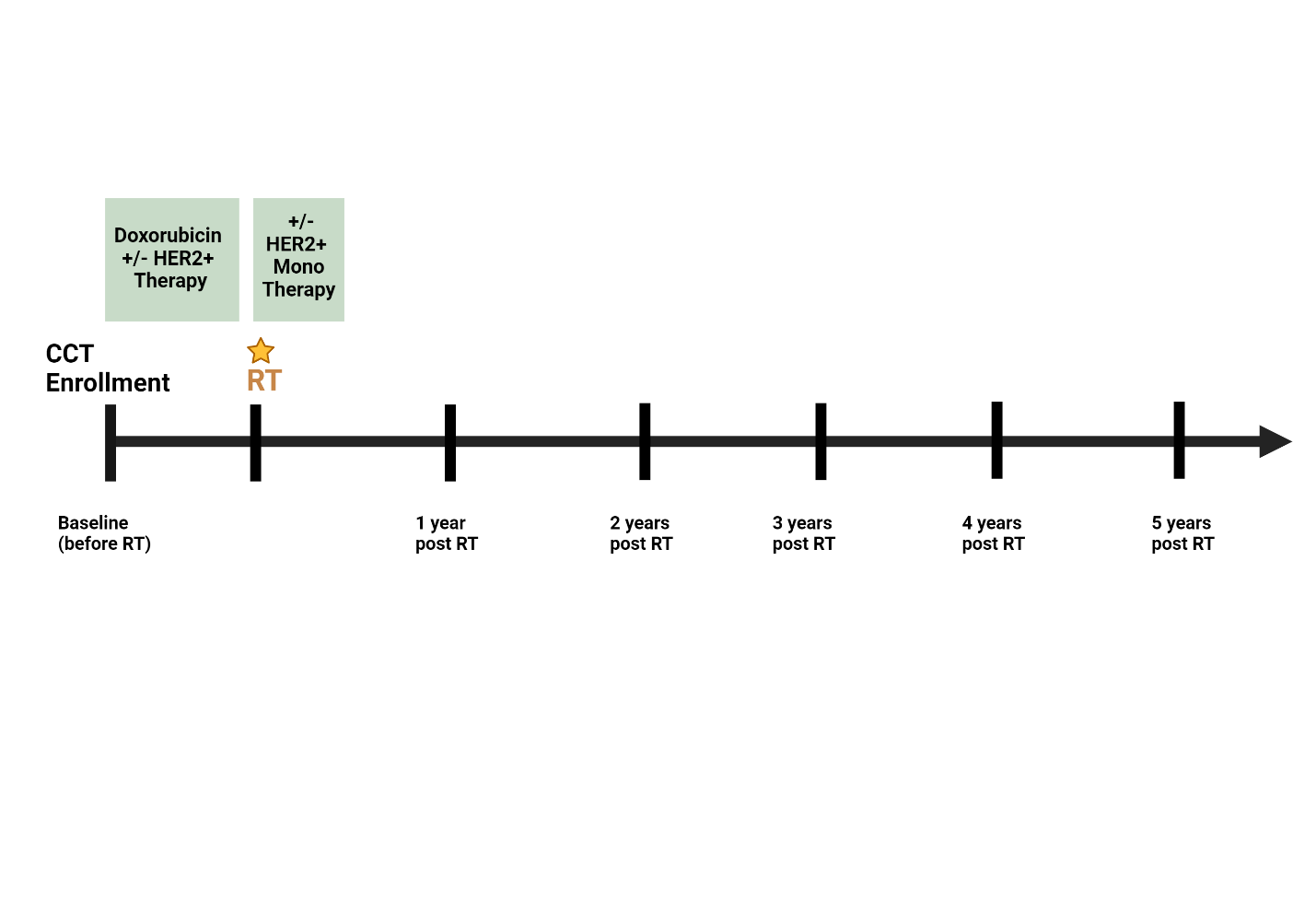
**

**Supplemental Figure 2: Change in Echocardiogram Parameters Over Time Stratified by Chemotherapy Regimen**

Plots are stratified by chemotherapy regimen and correspond to the predicted mean change in echocardiogram parameter over time from baseline (time = 0, pre-RT), and at 0.5, 1, 2, 3, 4 and 5 years and were estimated by GEE models. The model was adjusted for the following covariates: age, cancer stage (1 and 2 vs 3 and 4), body mass index, smoking status, systolic blood pressure, race, baseline hypertension, baseline diabetes, baseline cardioprotective medications (beta-blockers, angiotensin converting enzyme inhibitors, angiotensin receptor blockers, statins), time since last chemotherapy exposure to start of radiation, systemic cancer therapy (doxorubicin, trastuzumab, doxorubicin + trastuzumab), time since initiation of radiation, treatment site (left/bilateral, right), and baseline echocardiographic measure of interest. Lines within bars represent 95% CIs.

Along the y-axis, a bolded black arrow is placed to indicate a detriment in cardiovascular function relative to baseline.

Abbreviations: CI = confidence interval; RT = radiation therapy; LVEF = left ventricular ejection fraction; FAC = fractional area change


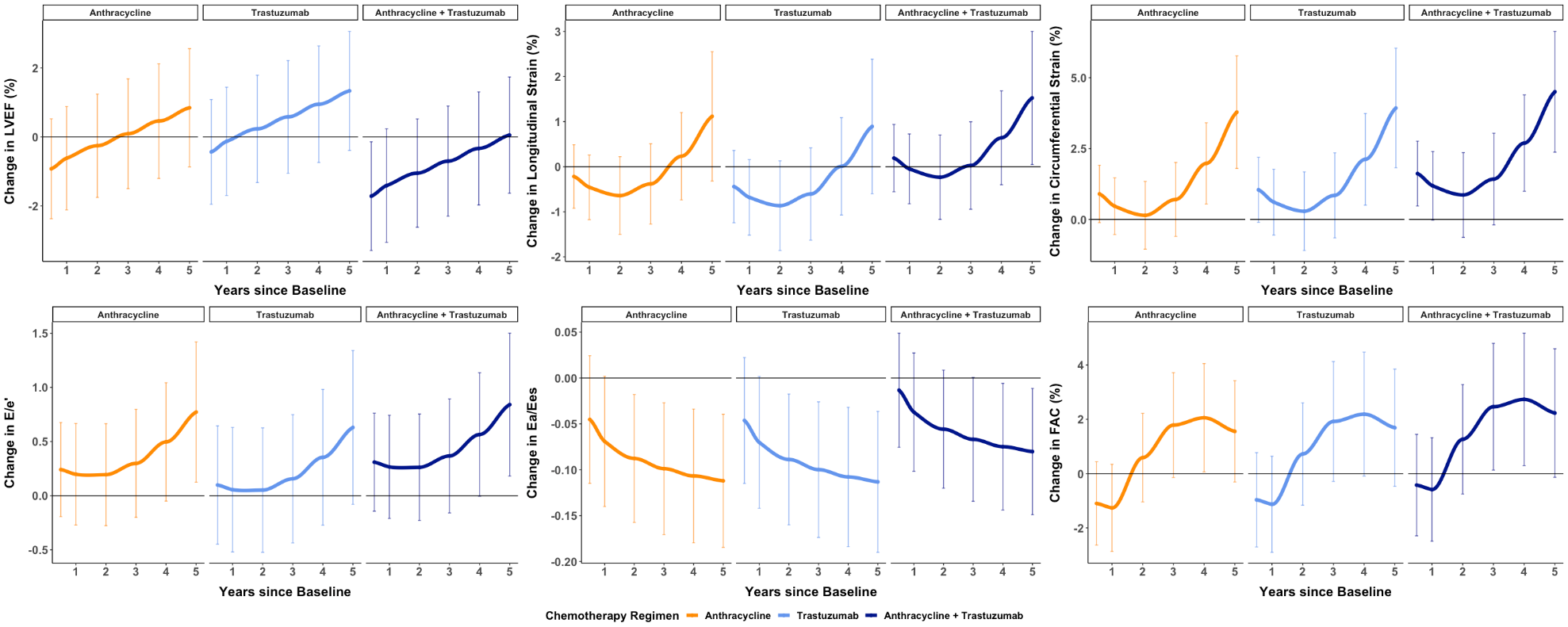


**Supplemental Table 1: Longitudinal Quantitative Echocardiography Measures Prior to, During, and After Radiation Therapy: for All Participants (A), Bilateral or Left Treatment Site (B), and Right Treatment Site (C)**

| **Supplemental Table 1A. All Participants** | | | | | | | | |
| --- | --- | --- | --- | --- | --- | --- | --- | --- |
|  | **Predicted mean (95% CI)** | | | | | | | ***p*-value** |
|  | **Baseline**  **pre RT** | **6 months post RT** | **1 year**  **post RT** | **2 years**  **post RT** | **3 years**  **post RT** | **4 years post RT** | **5 years post RT** |  |
| **LVEF**, % | 52.1  (50.8, 53.4) | 52.5  (51.1, 53.9) | 52.8  (51.4, 54.3) | 53.2  (51.8, 54.6) | 53.6  (52.1, 55.0) | 53.9  (52.4, 55.5) | 54.3  (52.7, 55.9) | < 0.001 |
| **Longitudinal strain**, % | -16.0  (-16.5, -15.4) | -16.1  (-16.7, -15.4) | -16.3  (-17.0, -15.6) | -16.5  (-17.3, -15.6) | -16.2  (-17.1, -15.3) | -15.6  (-16.6, -14.6) | -14.7  (-16.2, -13.3) | 0.089 |
| **Circumferential strain**, % | -23.7  (-24.6, -22.8) | -23.9  (-24.8, -22.9) | -24.3  (-25.3, -23.3) | -24.6  (-25.9, -23.4) | -24.1  (-25.4, -22.7) | -22.8  (-24.3, -21.3) | -21.0  (-23.0, -19.0) | 0.003 |
| **E/e’** | 8.6  (8.2, 9.0) | 8.5  (8.1, 9.0) | 8.5  (8.0, 9.0) | 8.5  (8.0, 9.0) | 8.6  (8.1, 9.1) | 8.8  (8.2, 9.3) | 9.1  (8.4, 9.7) | 0.073 |
| **Ea/Ees** | 1.02  (0.96, 1.08) | 0.98  (0.92, 1.04) | 0.96  (0.9, 1.02) | 0.94  (0.88, 1.00) | 0.93  (0.86, 0.99) | 0.92  (0.85, 0.99) | 0.92  (0.85, 0.98) | < 0.001 |
| **Right ventricular fractional area change,** % | 47.4  (45.8, 49.0) | 45.5  (44.0, 47.0) | 45.3  (43.8, 46.9) | 47.2  (45.6, 48.8) | 48.4  (46.4, 50.4) | 48.7  (46.6, 50.7) | 48.2  (46.2, 50.1) | 0.004 |
| Generalized estimating equations model was adjusted for the following covariates: age, cancer stage (1 and 2 versus 3 and 4), body mass index, smoking status, systolic blood pressure, race, baseline hypertension, baseline diabetes, baseline cardioprotective medications (beta-blockers, angiotensin converting enzyme inhibitors, angiotensin receptor blockers, statins), systemic cancer therapy (doxorubicin, trastuzumab, or doxorubicin + trastuzumab), time since last chemotherapy exposure to start of radiation, time since initiation of radiation, treatment site (left/bilateral, right), and baseline echocardiographic measure of interest.  Abbreviations: CI = confidence interval; RT = radiation therapy; LVEF = left ventricular ejection fraction | | | | | | | | |

| **Supplemental Table 1B. Bilateral or Left Treatment Sites** | | | | | | | | |
| --- | --- | --- | --- | --- | --- | --- | --- | --- |
|  | **Predicted mean (95% CI)** | | | | | | | ***p*-value** |
|  | **Baseline**  **pre RT** | **6 months**  **post RT** | **1 year**  **post RT** | **2 years**  **post RT** | **3 years**  **post RT** | **4 years post RT** | **5 years post RT** |  |
| **LVEF**, % | 51.9  (50.8, 53.0) | 53.0  (51.8, 54.2) | 53.5  (52.3, 54.8) | 53.6  (52.3, 54.9) | 53.6  (52.1, 55.0) | 53.5  (52.0, 55.1) | 53.6  (51.9, 55.2) | < 0.001 |
| **Longitudinal strain**, % | -15.7  (-16.4, -15.0) | -15.4  (-16.2, -14.6) | -15.9  (-16.8, -15.0) | -16.4  (-17.5, -15.3) | -16.0  (-17.2, -14.9) | -15.0  (-16.2, -13.9) | -13.6  (-14.8, -12.3) | < 0.001 |
| **Circumferential strain**, % | -23.4  (-24.3, -22.4) | -23.1  (-24.3, -22.0) | -23.9  (-25.1, -22.6) | -24.9  (-26.5, -23.3) | -24.3  (-26.1, -22.4) | -22.4  (-24.4, -20.4) | -19.7  (-22.0, -17.4) | < 0.001 |
| **E/e’** | 8.3  (7.9, 8.8) | 8.3  (7.8, 8.8) | 8.3  (7.8, 8.8) | 8.3  (7.7, 8.8) | 8.4  (7.8, 8.9) | 8.6  (8.0, 9.2) | 9.0  (8.3, 9.7) | 0.045 |
| **Ea/Ees** | 0.99  (0.94, 1.05) | 0.97  (0.91, 1.02) | 0.95  (0.89, 1.00) | 0.91  (0.86, 0.97) | 0.90  (0.83, 0.96) | 0.89  (0.82, 0.96) | 0.89  (0.82, 0.96) | 0.007 |
| **Right ventricular fractional area change,** % | 48.8  (46.9, 50.7) | 47.1  (45.3, 48.9) | 46.9  (45.0, 48.9) | 48.9  (47.0, 50.9) | 50.3  (47.9, 52.7) | 50.5  (48.0, 53.0) | 49.7  (47.4, 52.1) | 0.012 |
| Generalized estimating equations model was adjusted for the following covariates: age, cancer stage (1 and 2 versus 3 and 4), body mass index, smoking status, systolic blood pressure, race, baseline hypertension, baseline diabetes, baseline cardioprotective medications (beta-blockers, angiotensin converting enzyme inhibitors, angiotensin receptor blockers, statins), time since last chemotherapy exposure to start of radiation, systemic cancer therapy (doxorubicin, trastuzumab, or doxorubicin + trastuzumab), time since initiation of radiation, and baseline echocardiographic measure of interest.  Abbreviations: CI = confidence interval; RT = radiation therapy; LVEF = left ventricular ejection fraction | | | | | | | | |

| **Supplemental Table 1C. Right Treatment Site** | | | | | | | | |
| --- | --- | --- | --- | --- | --- | --- | --- | --- |
|  | **Predicted mean (95% CI)** | | | | | | | ***p*-value** |
|  | **Baseline**  **pre RT** | **6 months post RT** | **1 year**  **post RT** | **2 years**  **post RT** | **3 years**  **post RT** | **4 years post RT** | **5 years post RT** |  |
| **LVEF**, % | 52.4  (50.7, 54.1) | 52.1  (50.2, 54.0) | 52.2  (50.2, 54.2) | 53.1  (51.2, 54.9) | 54.0  (52.1, 55.9) | 54.9  (53.0, 56.9) | 55.8  (53.9, 57.7) | < 0.001 |
| **Longitudinal strain**, % | -16.2  (-16.9, -15.5) | -16.6  (-17.5, -15.7) | -16.6  (-17.5, -15.8) | -16.6  (-17.6, -15.6) | -16.5  (-17.5, -15.4) | -16.3  (-17.5, -15.1) | -16.1  (-17.9, -14.2) | 0.49 |
| **Circumferential strain**, % | -24.4  (-25.6, -23.2) | -25.0  (-26.4, -23.7) | -25.0  (-26.4, -23.6) | -24.6  (-26.3, -23.0) | -24.1  (-25.9, -22.2) | -23.4  (-25.3, -21.4) | -22.5  (-25.3, -19.8) | 0.17 |
| **E/e’** | 8.5  (7.9, 9.1) | 8.3  (7.8, 8.8) | 8.3  (7.7, 8.8) | 8.3  (7.8, 8.8) | 8.4  (7.8, 9.0) | 8.5  (7.9, 9.1) | 8.6  (7.9, 9.3) | 0.64 |
| **Ea/Ees** | 1.07  (0.98, 1.17) | 1.01  (0.91, 1.11) | 0.99  (0.88, 1.09) | 0.99  (0.89, 1.09) | 0.99  (0.88, 1.09) | 0.98  (0.87, 1.08) | 0.96  (0.86, 1.06) | < 0.001 |
| **Right ventricular fractional area change,** % | 47.4  (45.2, 49.7) | 45.5  (43.3, 47.6) | 45.3  (43.2, 47.5) | 46.9  (44.6, 49.1) | 47.9  (45.1, 50.7) | 48.3  (45.3, 51.4) | 48.3  (45.3, 51.3) | 0.15 |
| Generalized estimating equations model was adjusted for the following covariates: age, cancer stage (1 and 2 versus 3 and 4), body mass index, smoking status, systolic blood pressure, race, baseline hypertension, baseline diabetes, baseline cardioprotective medications (beta-blockers, angiotensin converting enzyme inhibitors, angiotensin receptor blockers, statins), time since last chemotherapy exposure to start of radiation, systemic cancer therapy (doxorubicin, trastuzumab, or doxorubicin + trastuzumab), time since initiation of radiation, and baseline echocardiographic measure of interest.  Abbreviations: CI = confidence interval; RT = radiation therapy; LVEF = left ventricular ejection fraction | | | | | | | | |

**Supplemental Table 2: Longitudinal Quantitative Echocardiography Measures Prior to, During, and After Radiation Therapy Stratified by Chemotherapy Regimen: Summary of Mean Heart Dose (A), Anthracycline (B), Trastuzumab (C), Anthracycline + Trastuzumab (D)**

| **Supplemental Table 2A. Summary of MHD by Chemotherapy Regimen** | | | | |
| --- | --- | --- | --- | --- |
|  | Anthracycline  N = 187^1^ | Trastuzumab  N = 51^1^ | Anthracycline + Trastuzumab  N = 65^1^ | *p*-value |
| MHD, Gy | 1.22  (0.76, 2.41) | 0.98  (0.54, 1.79) | 1.28  (0.81, 2.52) | 0.040 |
| ^1^ Median (Q1, Q3)  Abbreviations: MHD = mean heart dose, Gy = Gray | | | | |

| **Supplemental Table 2B. Anthracycline (N = 187)** | | | | | | | | |
| --- | --- | --- | --- | --- | --- | --- | --- | --- |
|  | **Baseline** | **6 months**  **post RT** | **1 year**  **post RT** | **2 years**  **post RT** | **3 years**  **post RT** | **4 years**  **post RT** | **5 years**  **post RT** | ***p*-value** |
| **LVEF, %** | 51.2  (49.1, 53.4) | 52.2  (50.0, 54.5) | 52.7  (50.36, 55.0) | 52.5  (50.2, 54.8) | 52.2  (49.8, 54.6) | 52.2  (49.8, 54.7) | 52.5  (50.0, 54.9) | 0.002 |
| **Longitudinal strain, %** | -16.2  (-17.1, -15.3) | -16.4  (-17.3, -15.4) | -16.5  (-17.5, -15.5) | -16.7  (-17.9, -15.5) | -16.5  (-17.7, -15.2) | -15.9  (-17.3, -14.5) | -15.1  (-17.1, -13.1) | 0.25 |
| **Circumferential strain, %** | -23.9  (-24.9, -22.8) | -24.3  (-25.5, -23.2) | -24.6  (-25.8, -23.3) | -24.5  (-25.9, -23.0) | -23.8  (-25.4, -22.3) | -22.8  (-24.5, -21.1) | -21.5  (-23.9, -19.2) | 0.042 |
| **E/e’** | 8.7  (8.2, 9.2) | 8.6  (8.1, 9.2) | 8.6  (8.0, 9.1) | 8.5  (7.9, 9.1) | 8.6  (8.0, 9.2) | 8.8  (8.1, 9.5) | 9.1  (8.3, 9.9) | 0.10 |
| **Ea/Ees** | 1.08  (0.98, 1.17) | 1.03  (0.93, 1.13) | 1.01  (0.90, 1.11) | 1.00  (0.89, 1.11) | 1.00  (0.89, 1.11) | 0.99  (0.88, 1.10) | 0.98  (0.88, 1.09) | < 0.001 |
| **Right ventricular fractional area change,%** | 47.3  (45.3, 49.3) | 45.3  (43.5, 47.1) | 44.7  (42.9, 46.6) | 46.5  (44.7, 48.3) | 47.9  (45.6, 50.2) | 48.4  (45.9, 50.9) | 48.2  (45.8, 50.6) | 0.022 |
| *^1^* predicted mean (95% CI)  Generalized estimating equations model was adjusted for the following covariates: age, cancer stage (1 and 2 versus 3 and 4), body mass index, smoking status, systolic blood pressure, race, baseline hypertension, baseline diabetes, baseline cardioprotective medications (beta-blockers, angiotensin converting enzyme inhibitors, angiotensin receptor blockers, statins), time since last chemotherapy exposure to start of radiation, time since initiation of radiation, treatment site (left/bilateral, right), and baseline echocardiographic measure of interest.  Abbreviations: CI = confidence interval; LVEF = left ventricular ejection fraction; RV = right ventricular; FAC = fractional area change | | | | | | | | |

| **Supplemental Table 2C. Trastuzumab (N = 51)** | | | | | | | | |
| --- | --- | --- | --- | --- | --- | --- | --- | --- |
|  | **Baseline** | **6 months**  **post RT** | **1 year**  **post RT** | **2 years**  **post RT** | **3 years**  **post RT** | **4 years**  **post RT** | **5 years**  **post RT** | ***p*-value** |
| **LVEF, %** | 54.5  (52.5, 56.5) | 54.6  (52.5, 56.8) | 55.1  (53.0, 57.2) | 56.0  (53.7, 58.3) | 56.8  (54.3, 59.3) | 57.5  (54.9, 60.2) | 58.2  (55.5, 60.9) | < 0.001 |
| **Longitudinal strain, %** | -16.8  (-18.2, -15.4) | -17.2  (-18.9, -15.5) | -17.2  (-19.1, -15.4) | -17.2  (-19.4, -15.1) | -17.1  (-19.2, -15.0) | -16.8  (-19.0, -14.6) | -16.5  (-19.2, -13.8) | 0.76 |
| **Circumferential strain, %** | -26.8  (-28.1, -25.4) | -26.2  (-27.9, -24.5) | -27.3  (-29.1, -25.4) | -27.7  (-29.9, -25.4) | -26.4  (-29.3, -23.4) | -23.9  (-29.2, -18.6) | -21.0  (-29.5, -12.5) | 0.19 |
| **E/e’** | 9.8  (9.1, 10.5) | 9.7  (8.9, 10.5) | 9.7  (8.9, 10.6) | 10.0  (9.2, 10.8) | 10.2  (9.3, 11.0) | 10.3  (9.5, 11.2) | 10.5  (9.6, 11.3) | 0.057 |
| **Ea/Ees** | 0.81  (0.72, 0.91) | 0.80  (0.70, 0.90) | 0.76  (0.67, 0.85) | 0.70  (0.61, 0.80) | 0.68  (0.58, 0.78) | 0.69  (0.58, 0.79) | 0.72  (0.60, 0.83) | 0.034 |
| **Right ventricular fractional area change,%** | 44.0  (41.7, 46.3) | 42.9  (40.6, 45.1) | 43.6  (41.6, 45.7) | 44.8  (41.9, 47.7) | 45.1  (41.6, 48.6) | 44.8  (41.4, 48.2) | 43.9  (41.0, 46.8) | 0.16 |
| *^1^* predicted mean (95% CI)  Generalized estimating equations model was adjusted for the following covariates: age, cancer stage (1 and 2 versus 3 and 4), body mass index, smoking status, systolic blood pressure, race, baseline hypertension, baseline diabetes, baseline cardioprotective medications (beta-blockers, angiotensin converting enzyme inhibitors, angiotensin receptor blockers, statins), time since last chemotherapy exposure to start of radiation, time since initiation of radiation, treatment site (left/bilateral, right), and baseline echocardiographic measure of interest.  Abbreviations: CI = confidence interval; LVEF = left ventricular ejection fraction; RV = right ventricular; FAC = fractional area change | | | | | | | | |

| **Supplemental Table 2D. Anthracycline + Trastuzumab (N = 65)** | | | | | | | | |
| --- | --- | --- | --- | --- | --- | --- | --- | --- |
|  | **Baseline** | **6 months**  **post RT** | **1 year**  **post RT** | **2 years**  **post RT** | **3 years**  **post RT** | **4 years**  **post RT** | **5 years post RT** | ***p*-value** |
| **LVEF, %** | 52.1  (49.9, 54.4) | 51.6  (49.4, 53.7) | 51.9  (49.7, 54.1) | 53.2  (50.9, 55.5) | 54.4  (51.8, 57.0) | 55.4  (52.6, 58.2) | 56.3  (53.5, 59.2) | 0.003 |
| **Longitudinal strain, %** | 15.8  (-16.5, -15.2) | -15.7  (-16.6, -14.8) | -16.1  (-16.9, -15.3) | -16.3  (-17.4, -15.2) | -16.0  (-17.2, -14.8) | -15.2  (-16.3, -14.1) | -14.1  (-15.3, -12.9) | < 0.001 |
| **Circumferential strain, %** | -21.2  (-23.0, -19.4) | -20.8  (-22.6, -18.9) | -21.8  (-23.7, -19.9) | -22.9  (-25.6, -20.1) | -22.2  (-25.4, -19.0) | -20.4  (-23.7, -17.1) | -17.7  (-21.1, -14.3) | < 0.001 |
| **E/e’** | 7.9  (7.4, 8.4) | 7.9  (7.5, 8.3) | 7.9  (7.5, 8.3) | 7.8  (7.3, 8.3) | 7.8  (7.2, 8.5) | 7.9  (7.2, 8.6) | 8.0  (7.3, 8.8) | 0.77 |
| **Ea/Ees** | 1.06  (0.98, 1.14) | 1.03  (0.95, 1.10) | 1.00  (0.92, 1.08) | 0.96  (0.87, 1.05) | 0.93  (0.82, 1.04) | 0.92  (0.81, 1.04) | 0.93  (0.82, 1.04) | 0.25 |
| **Right**  **ventricular fractional area change,%** | 43.7  (41.7, 45.7) | 42.1  (40.1, 44.0) | 42.8  (40.7, 44.9) | 44.8  (41.3, 48.3) | 45.1  (41.1, 49.1) | 43.9  (40.7, 47.2) | 41.6  (38.7, 44.5) | 0.27 |
| *^1^* predicted mean (95% CI)  Generalized estimating equations model was adjusted for the following covariates: age, cancer stage (1 and 2 versus 3 and 4), body mass index, smoking status, systolic blood pressure, race, baseline hypertension, baseline diabetes, baseline cardioprotective medications (beta-blockers, angiotensin converting enzyme inhibitors, angiotensin receptor blockers, statins), time since last chemotherapy exposure to start of radiation, time since initiation of radiation, treatment site (left/bilateral, right), and baseline echocardiographic measure of interest.  Abbreviations: CI = confidence interval; LVEF = left ventricular ejection fraction; RV = right ventricular; FAC = fractional area change | | | | | | | | |

**Supplemental Table 3: Association between Radiation Dose Metrics and Cardiac Function Parameters Stratified by Chemotherapy Regimen Across All Timepoints: For MHD (A), LAD Maximum Dose (B), Mean LV Dose (C), Mean RV Dose (D)**

| **Supplemental Table 3A. MHD** | | | | | | |
| --- | --- | --- | --- | --- | --- | --- |
|  | **Anthracycline** | | **Trastuzumab** | | **Anthracycline + Trastuzumab** | |
|  | $\boldsymbol{\beta}$**(95% CI)** | ***p*-value** | $\boldsymbol{\beta}$ **(95% CI)** | ***p*-value** | $\boldsymbol{\beta}$ **(95% CI)** | ***p*-value** |
| **LVEF, %** | -0.70  (-1.68, 0.29) | 0.16 | -0.88  (-1.94, 0.18) | 0.10 | -0.36  (-1.55, 0.83) | 0.55 |
| **Longitudinal strain, %** | 0.61  (0.14, 1.08) | 0.011 | -0.33  (-1.04, 0.38) | 0.36 | 0.76  (0.52, 0.99) | < 0.001 |
| **Circumferential strain, %** | 0.28  (-0.44, 1.01) | 0.45 | 0.27  (-0.65, 1.19) | 0.57 | -0.50  (-1.17, 0.17) | 0.14 |
| **E/e’** | 0.20  (-0.12, 0.53) | 0.22 | 0.26  (0.00, 0.52) | 0.053 | -0.07  (-0.24, 0.11) | 0.46 |
| **Ea/Ees** | 0.02  (-0.04, 0.07) | 0.55 | 0.05  (-0.02, 0.11) | 0.16 | -0.01  (-0.04, 0.03) | 0.68 |
| **Right ventricular fractional area change,%** | -0.51  (-1.62, 0.60) | 0.37 | -0.56  (-1.64, 0.51) | 0.30 | 1.32  (0.30, 2.34) | 0.012 |
| Generalized estimating equations model was adjusted for the following covariates: age, cancer stage (1, 2 versus 3, 4), body mass index, smoking status, systolic blood pressure, race, baseline hypertension, baseline diabetes, baseline cardioprotective medications (beta-blockers, angiotensin converting enzyme inhibitors, angiotensin receptor blockers, statins), time since last chemotherapy exposure to start of radiation, time since initiation of radiation, treatment site (left/bilateral, right), and baseline echocardiographic measure of interest.  $\beta$: coefficient estimates for the IQR standardized MHD  Abbreviations: CI = confidence interval; IQR = interquartile range; MHD = mean heart dose; LVEF = left ventricular ejection fraction; RV = right ventricular; FAC = fractional area change | | | | | | |

| **Supplemental Table 3B. LAD Maximum Dose** | | | | | | |
| --- | --- | --- | --- | --- | --- | --- |
|  | **Anthracycline** | | **Trastuzumab** | | **Anthracycline + Trastuzumab** | |
|  | $\boldsymbol{\beta}$ **(95% CI)** | ***p*-value** | $\boldsymbol{\beta}$ **(95% CI)** | ***p*-value** | $\boldsymbol{\beta}$ **(95% CI)** | ***p*-value** |
| **LVEF, %** | -3.29  (-4.78, -1.80) | < 0.001 | -5.18  (-8.33, -2.03) | 0.001 | 1.29  (-0.61, 3.18) | 0.18 |
| **Longitudinal strain, %** | 1.93  (0.91, 2.95) | < 0.001 | -0.49  (-2.33, 1.34) | 0.60 | 1.03  (-0.49, 2.55) | 0.18 |
| **Circumferential strain, %** | 2.24  (0.62, 3.85) | 0.007 | 0.74  (-1.30, 2.78) | 0.48 | -1.37  (-3.51, 0.77) | 0.21 |
| **E/e’** | 0.65  (-0.02, 1.33) | 0.059 | 1.30  (0.12, 2.48) | 0.031 | -0.07  (-0.65, 0.50) | 0.81 |
| **Ea/Ees** | 0.05  (-0.01, 0.11) | 0.082 | 0.20  (0.04, 0.36) | 0.012 | -0.08  (-0.17, 0.00) | 0.052 |
| **Right ventricular fractional area change,%** | -1.75  (-4.15, 0.64) | 0.15 | -1.67  (-5.94, 2.61) | 0.44 | 6.63  (3.67, 9.59) | < 0.001 |
| Generalized estimating equations model was adjusted for the following covariates: age, cancer stage (1, 2 versus 3, 4), body mass index, smoking status, systolic blood pressure, race, baseline hypertension, baseline diabetes, baseline cardioprotective medications (beta-blockers, angiotensin converting enzyme inhibitors, angiotensin receptor blockers, statins), time since last chemotherapy exposure to start of radiation, time since initiation of radiation, treatment site (left/bilateral versus right), and baseline echocardiographic measure of interest.  $\beta$: coefficient estimates for the IQR standardized MHD  Abbreviations: CI = confidence interval; IQR = interquartile range; MHD = mean heart dose; LVEF = left ventricular ejection fraction; RV = right ventricular; FAC = fractional area change | | | | | | |

| **Supplemental Table 3C. Mean LV Dose** | | | | | | |
| --- | --- | --- | --- | --- | --- | --- |
|  | **Anthracycline** | | **Trastuzumab** | | **Anthracycline + Trastuzumab** | |
|  | $\boldsymbol{\beta}$ **(95% CI)** | ***p*-value** | $\boldsymbol{\beta}$ **(95% CI)** | ***p*-value** | $\boldsymbol{\beta}$ **(95% CI)** | ***p*-value** |
| **LVEF, %** | -1.11  (-2.16, -0.06) | 0.038 | -1.51  (-2.77, -0.25) | 0.019 | 0.46  (-0.70, 1.61) | 0.44 |
| **Longitudinal strain, %** | 0.75  (0.13, 1.37) | 0.018 | -0.50  (-1.48, 0.49) | 0.32 | 1.04  (0.54, 1.53) | < 0.001 |
| **Circumferential strain, %** | 0.50  (-0.40, 1.40) | 0.28 | 0.39  (-0.75, 1.54) | 0.50 | -0.66  (-1.54, 0.23) | 0.15 |
| **E/e’** | 0.27  (-0.10, 0.63) | 0.16 | 0.41  (0.01, 0.81) | 0.047 | -0.12  (-0.38, 0.14) | 0.37 |
| **Ea/Ees** | 0.01  (-0.05, 0.06) | 0.80 | 0.08  (-0.01, 0.17) | 0.074 | -0.03  (-0.07, 0.02) | 0.30 |
| **Right ventricular fractional area change,%** | -0.61  (-1.73, 0.51) | 0.29 | -1.16  (-2.52, 0.19) | 0.093 | 1.60  (0.19, 3.02) | 0.026 |
| Generalized estimating equations model was adjusted for the following covariates: age, cancer stage (1, 2 versus 3, 4), body mass index, smoking status, systolic blood pressure, race, baseline hypertension, baseline diabetes, baseline cardioprotective medications (beta-blockers, angiotensin converting enzyme inhibitors, angiotensin receptor blockers, statins), time since last chemotherapy exposure to start of radiation, time since initiation of radiation, treatment site (left/bilateral versus right), and baseline echocardiographic measure of interest.  $\beta$: coefficient estimates for the IQR standardized MHD  Abbreviations: CI = confidence interval; IQR = interquartile range; MHD = mean heart dose; LVEF = left ventricular ejection fraction; RV = right ventricular; FAC = fractional area change | | | | | | |

| **Supplemental Table 3D. Mean RV Dose** | | | | | | |
| --- | --- | --- | --- | --- | --- | --- |
|  | **Anthracycline** | | **Trastuzumab** | | **Anthracycline + Trastuzumab** | |
|  | $\boldsymbol{\beta}$ **(95% CI)** | ***p*-value** | $\boldsymbol{\beta}$ **(95% CI)** | ***p*-value** | $\boldsymbol{\beta}$ **(95% CI)** | ***p*-value** |
| **LVEF, %** | -0.03  (-0.55, 0.49) | 0.91 | -0.20  (-0.72, 0.33) | 0.47 | -0.22  (-0.76, 0.32) | 0.42 |
| **Longitudinal strain, %** | 0.21  (-0.02, 0.43) | 0.070 | -0.20  (-0.52, 0.13) | 0.24 | 0.35  (0.27, 0.44) | < 0.001 |
| **Circumferential strain, %** | 0.14  (-0.28, 0.56) | 0.51 | 0.05  (-0.46, 0.56) | 0.85 | -0.52  (-0.93, -0.11) | 0.013 |
| **E/e’** | 0.07  (-0.13, 0.27) | 0.48 | 0.13  (-0.01, 0.27) | 0.070 | -0.04  (-0.11, 0.03) | 0.26 |
| **Ea/Ees** | 0.01  (-0.02, 0.03) | 0.53 | 0.00  (-0.02, 0.03) | 0.78 | -0.01  (-0.02, 0.01) | 0.43 |
| **Right ventricular fractional area change,%** | 0.03  (-0.63, 0.69) | 0.93 | 0.13  (-0.49, 0.75) | 0.68 | 0.50  (0.12, 0.89) | 0.011 |
| Generalized estimating equations model was adjusted for the following covariates: age, cancer stage (1, 2 versus 3, 4), body mass index, smoking status, systolic blood pressure, race, baseline hypertension, baseline diabetes, baseline cardioprotective medications (beta-blockers, angiotensin converting enzyme inhibitors, angiotensin receptor blockers, statins), time since last chemotherapy exposure to start of radiation, time since initiation of radiation, treatment site (left/bilateral versus right), and baseline echocardiographic measure of interest.  $\beta$: coefficient estimates for the IQR standardized MHD  Abbreviations: CI = confidence interval; IQR = interquartile range; MHD = mean heart dose; LVEF = left ventricular ejection fraction; RV = right ventricular; FAC = fractional area change | | | | | | |

**Supplemental Table 4: Mean Echocardiographic Measures (95% CI) for Bilateral or Left Breast Chest Wall Patients For: Mean LAD dose ≥/< 10 Gy (A), Mean LV dose ≥/< 3 Gy (B), LV V5 dose ≥/< 17% (C)**

| **Supplemental Table 4A: Mean LAD dose ≥/< 10 Gy** | | | | | | | | | |
| --- | --- | --- | --- | --- | --- | --- | --- | --- | --- |
|  |  | **Baseline** | **6 months**  **post RT** | **1 year**  **post RT** | **2 years**  **post RT** | **3 years**  **post RT** | **4 years**  **post RT** | **5 years**  **post RT** | ***p-*value** |
| **≥ 10 Gy** | **LVEF (%)** | 51.0  (49.5, 52.4) | 52.2  (50.7, 53.6) | 52.8  (51.2, 54.4) | 52.8  (51.2, 54.4) | 52.5  (50.8, 54.3) | 52.5  (50.6, 54.4) | 52.6  (50.7, 54.6) | 0.27 |
| **< 10 Gy** | **LVEF (%)** | 53.7  (52.2, 55.2) | 54.4  (52.6, 56.1) | 55.1  (53.5, 56.7) | 55.9  (54.2, 57.5) | 55.9  (53.9, 57.8) | 55.3  (53.1, 57.4) | 54.2  (51.6, 56.7) |  |
| **≥ 10 Gy** | **Longitudinal strain (%)** | -15.9  (-16.6, -15.1) | -15.6  (-16.5, -14.7) | -15.8  (-16.8, -14.7) | -16.3  (-17.5, -15.2) | -16.1  (-17.3, -14.8) | -15.1  (-16.3, -13.9) | -13.7  (-14.9, -12.5) | 0.027 |
| **< 10 Gy** | **Longitudinal strain (%)** | -16.0  (-17.0, -15.1) | -15.8  (-16.7, -14.8) | -16.7  (-17.8, -15.5) | -17.6  (-19.3, -15.8) | -17.5  (-19.4, -15.6) | -16.8  (-18.9, -14.8) | -15.8  (-18.5, -13.0) |  |
| **≥ 10 Gy** | **Circumferential strain (%)** | -23.9  (-25.0, -22.8) | -23.5  (-24.6, -22.5) | -23.9  (-25.1, -22.7) | -25.0  (-26.7, -23.3) | -24.5  (-26.6, -22.4) | -22.6  (-24.7, -20.4) | -19.7  (-22.1, -17.4) | 0.15 |
| **< 10 Gy** | **Circumferential strain (%)** | -23.8  (-25.9, -21.6) | -23.6  (-26.3, -20.9) | -25.3  (-28.2, -22.4) | -27.0  (-30.4, -23.6) | -26.9  (-30.2, -23.5) | -25.4  (-28.8, -22.0) | -23.3  (-27.9, -18.8) |  |
| **≥ 10 Gy** | **E/e’** | 8.3  (7.8, 8.7) | 8.2  (7.7, 8.7) | 8.2  (7.6, 8.8) | 8.2  (7.6, 8.8) | 8.3  (7.7, 9) | 8.6  (7.9, 9.2) | 8.9  (8.2, 9.6) | 0.42 |
| **< 10 Gy** | **E/e’** | 8.7  (8.1, 9.4) | 8.8  (8.1, 9.5) | 8.8  (8.1, 9.5) | 8.6  (7.9, 9.3) | 8.7  (8, 9.5) | 9.0  (7.9, 10.0) | 9.4  (7.7, 11.2) |  |
| **≥ 10 Gy** | **Ea/Ees** | 1.10  (0.99, 1.14) | 1.03  (0.96, 1.11) | 1.01  (0.93, 1.09) | 0.97  (0.9, 1.05) | 0.95  (0.87, 1.04) | 0.95  (0.86, 1.03) | 0.95  (0.86, 1.03) | 0.042 |
| **< 10 Gy** | **Ea/Ees** | 0.90  (0.82, 0.97) | 0.88  (0.81, 0.96) | 0.85  (0.78, 0.93) | 0.81  (0.72, 0.89) | 0.79  (0.69, 0.88) | 0.79  (0.69, 0.89) | 0.81  (0.70, 0.92) |  |
| **≥ 10 Gy** | **RV FAC (%)** | 48.9  (46.8, 51) | 45.6  (43.6, 47.6) | 44.4  (42.1, 46.7) | 47.0  (44.7, 49.3) | 50.6  (48, 53.1) | 52.0  (49.3, 54.7) | 51.3  (48.7, 53.9) | 0.48 |
| **< 10 Gy** | **RV FAC (%)** | 44.3  (39.8, 48.9) | 47.6  (43.1, 52.1) | 46.6  (42.4, 50.8) | 44.6  (40, 49.2) | 43.5  (38.4, 48.5) | 43.0  (37.9, 48.2) | 43.1  (38.1, 48.1) |  |

| **Supplemental Table 4B: Mean LV dose ≥/< 3 Gy** | | | | | | | | | |
| --- | --- | --- | --- | --- | --- | --- | --- | --- | --- |
|  |  | **Baseline** | **6 months**  **post RT** | **1 year**  **post RT** | **2 years**  **post RT** | **3 years**  **post RT** | **4 years**  **post RT** | **5 years**  **post RT** | ***p-*value** |
| **≥ 3 Gy** | **LVEF (%)** | 49.9  (48.5, 51.3) | 51.2  (49.8, 52.5) | 51.9  (50.4, 53.4) | 51.7  (50.2, 53.2) | 51.0  (49.5, 52.6) | 50.8  (49.1, 52.5) | 50.9  (49.1, 52.7) | 0.052 |
| **< 3 Gy** | **LVEF (%)** | 54.2  (52.9, 55.4) | 54.7  (53.2, 56.2) | 55.3  (53.9, 56.7) | 56.3  (54.7, 57.9) | 56.9  (55.1, 58.8) | 57.2  (55.1, 59.4) | 57.2  (54.4, 60.1) |  |
| **≥ 3 Gy** | **Longitudinal strain (%)** | -15.3  (-16.1, -14.4) | -15.2  (-16.0, -14.3) | -15.3  (-16.2, -14.3) | -15.6  (-16.7, -14.4) | -15.2  (-16.6, -13.9) | -14.4  (-15.6, -13.1) | -13.1  (-14.5, -11.8) | 0.10 |
| **< 3 Gy** | **Longitudinal strain (%)** | -16.3  (-17.1, -15.5) | -15.8  (-16.8, -14.9) | -16.8  (-17.9, -15.7) | -17.9  (-19.5, -16.3) | -17.8  (-19.5, -16.1) | -16.9  (-18.5, -15.3) | -15.5  (-17.3, -13.7) |  |
| **≥ 3 Gy** | **Circumferential strain (%)** | -23.8  (-25.1, -22.6) | -23.4  (-24.7, -22.1) | -23.7  (-25.2, -22.2) | -24.8  (-26.7, -23.0) | -24.3  (-26.5, -22.0) | -22.2  (-24.6, -19.9) | -19.2  (-21.9, -16.6) | 0.049 |
| **< 3 Gy** | **Circumferential strain (%)** | -23.4  (-24.7, -22.1) | -23.2  (-24.9, -21.6) | -24.7  (-26.3, -23.0) | -26.1  (-28.3, -24.0) | -26.1  (-28.3, -23.9) | -24.8  (-27.0, -22.7) | -22.9  (-25.5 -20.4) |  |
| **≥ 3 Gy** | **E/e’** | 8.2  (7.7, 8.6) | 8.4  (7.9, 8.8) | 8.4  (7.9, 9.0) | 8.4  (7.9, 9.0) | 8.4  (7.8, 9.0) | 8.6  (7.9, 9.2) | 8.9  (8.2, 9.6) | 0.098 |
| **< 3 Gy** | **E/e’** | 8.6  (8, 9.2) | 8.4  (7.7, 9.1) | 8.3  (7.5, 9.1) | 8.3  (7.5, 9.1) | 8.4  (7.6, 9.2) | 8.6  (7.6, 9.5) | 8.8  (7.6, 10.0) |  |
| **≥ 3 Gy** | **Ea/Ees** | 1.13  (1.03, 1.23) | 1.09  (1, 1.19) | 1.06  (0.96, 1.17) | 1.03  (0.93, 1.13) | 1.01  (0.91, 1.11) | 1.00  (0.89, 1.11) | 1.01  (0.90, 1.11) | 0.024 |
| **< 3 Gy** | **Ea/Ees** | 0.88  (0.83, 0.94) | 0.86  (0.81, 0.91) | 0.84  (0.79, 0.89) | 0.80  (0.75, 0.86) | 0.78  (0.72, 0.85) | 0.78  (0.71, 0.85) | 0.79  (0.72, 0.86) |  |
| **≥ 3 Gy** | **RV FAC (%)** | 49.4  (47.1, 51.7) | 46.2  (44.3, 48.2) | 45.0  (42.8, 47.2) | 47.4  (45.0, 49.7) | 51.3  (48.6, 54.0) | 52.9  (50.0, 55.8) | 52.4  (49.7, 55.1) | 0.095 |
| **< 3 Gy** | **RV FAC (%)** | 46.3  (43.6, 49.0) | 47.5  (44.5, 50.5) | 47.4  (44.6, 50.1) | 46.9  (43.6, 50.3) | 46.4  (42.5, 50.3) | 45.8  (41.8, 49.7) | 45.0  (41.6, 48.5) |  |

| **Supplemental Table 4C: LV V5 dose ≥/< 17%** | | | | | | | | | |
| --- | --- | --- | --- | --- | --- | --- | --- | --- | --- |
|  |  | **Baseline** | **6 months post RT** | **1 year**  **post RT** | **2 years**  **post RT** | **3 years**  **post RT** | **4 years**  **post RT** | **5 years**  **post RT** | ***p*-value** |
| **≥ 17%** | **LVEF (%)** | 50.7  (49.1, 52.3) | 51.8  (50.3, 53.3) | 52.3  (50.6, 54.0) | 52.1  (50.3, 53.8) | 51.3  (49.5, 53.0) | 51.0  (49.1, 52.9) | 51.2  (49.2, 53.2) | 0.068 |
| **< 17%** | **LVEF (%)** | 53.3  (52.2, 54.4) | 54.2  (52.9, 55.5) | 54.7  (53.4, 56.1) | 55.5  (54.0, 56.9) | 55.9  (54.1, 57.6) | 56.0  (54.0, 58.0) | 56.0  (53.9, 58.0) |  |
| **≥ 17%** | **Longitudinal strain (%)** | -15.2  (-15.8, -14.5) | -15.0  (-15.8, -14.1) | -15.1  (-16.1, -14.1) | -15.4  (-16.5, -14.3) | -15.0  (-16.4, -13.6) | -13.9  (-15.3, -12.6) | -12.4  (-13.9, -11.0) | 0.14 |
| **< 17%** | **Longitudinal strain (%)** | -15.9  (-16.7, -15.2) | -15.8  (-16.6, -14.9) | -16.5  (-17.5, -15.5) | -17.2  (-18.7, -15.8) | -17.1  (-18.5, -15.6) | -16.2  (-17.5, -14.9) | -14.9  (-16.3, -13.5) |  |
| **≥ 17%** | **Circumferential strain (%)** | -24.9  (-26.5, -23.3) | -24.6  (-26.1, -23.1) | -24.8  (-26.4, -23.2) | -25.7  (-27.3, -24.0) | -25.0  (-27.0, -23.0) | -22.9  (-25.1, -20.7) | -19.8  (-22.4, -17.3) | 0.30 |
| **< 17%** | **Circumferential strain (%)** | -23.1  (-24.4, -21.7) | -22.6  (-24.2, -21.1) | -24.0  (-25.7, -22.2) | -25.3  (-27.9, -22.6) | -24.7  (-27.7, -21.8) | -22.9  (-26.0, -19.8) | -20.3  (-23.8, -16.8) |  |
| **≥ 17%** | **E/e’** | 8.6  (8.0, 9.2) | 8.7  (8.0, 9.5) | 8.8  (7.9, 9.7) | 8.8  (7.8, 9.7) | 8.7  (7.7, 9.7) | 8.8  (7.9, 9.7) | 9.1  (8.2, 9.9) | 0.26 |
| **< 17%** | **E/e’** | 8.2  (7.7, 8.7) | 8.1  (7.5, 8.6) | 8.0  (7.4, 8.6) | 8.0  (7.4, 8.6) | 8.2  (7.5, 8.8) | 8.5  (7.6, 9.3) | 8.9  (7.8, 9.9) |  |
| **≥ 17%** | **Ea/Ees** | 1.17  (1.06, 1.29) | 1.14  (1.03, 1.25) | 1.12  (1.00, 1.23) | 1.06  (0.95, 1.18) | 1.03  (0.91, 1.14) | 1.02  (0.90, 1.13) | 1.03  (0.91, 1.14) | 0.21 |
| **< 17%** | **Ea/Ees** | 0.94  (0.89, 0.99) | 0.91  (0.86, 0.96) | 0.89  (0.83, 0.94) | 0.87  (0.81, 0.92) | 0.85  (0.78, 0.92) | 0.84  (0.77, 0.92) | 0.84  (0.75, 0.93) |  |
| **≥ 17%** | **RV FAC (%)** | 44.9  (42.9, 47.0) | 43.6  (41.8, 45.5) | 43.7  (41.3, 46.1) | 47.5  (45.4, 49.7) | 52.1  (50.0, 54.3) | 53.7  (51.4, 56.0) | 52.3  (50.4, 54.3) | 0.81 |
| **< 17%** | **RV FAC (%)** | 48.4  (46.0, 50.7) | 47.3  (45.0, 49.6) | 46.9  (44.5, 49.3) | 46.9  (44.6, 49.2) | 46.8  (44.0, 49.6) | 46.5  (43.5, 49.5) | 46.1  (43.2, 49.0) |  |
